# Propan-2-yl *r*-4-(4-fluoro­phen­yl)-3-hy­droxy-*c*-6-methyl-2-phenyl-4,5-dihydro-2*H*-indazole-*t*-5-carboxyl­ate

**DOI:** 10.1107/S1600536812039955

**Published:** 2012-09-26

**Authors:** S. Rizwana Begum, R. Hema, K. Pandiarajan, Sridhar Balasubramanian, A. G. Anitha

**Affiliations:** a Department of Physics, Seethalakshmi Ramaswami College (Autonomous), Tiruchirappalli 620 002, India; b Department of Chemistry, Annamalai University, Annamalai Nagar 608 002, India; c Laboratory of X-ray Crystallography, Indian Institute of Chemical Technology, Hyderabad 500 007, India

## Abstract

In the title compound, C_24_H_23_FN_2_O_3_, the cyclo­hexene ring adopts a screw-boat conformation. The fluorobenzene ring attached to the cyclo­hexene ring and the phenyl ring attached to the indazole moiety are inclined to one another by 57.77 (13)°. In the crystal, mol­ecules are linked by O—H⋯N and C—H⋯O hydrogen bonds, forming chains with *C*(5) and *C*(10) graph-set motifs. There are also C—H⋯π inter­actions present. The isopropoxycarbonyl group undergoes considerable thermal motion.

## Related literature

For examples of the biological activities of indazole derivatatives, see: Jain *et al.* (1987 ▶); Palazzo *et al.* (1966 ▶); Popat *et al.* (2003 ▶); Beylin *et al.* (1991 ▶); George *et al.* (1998 ▶); Roman (1990 ▶). For the crystal structure of a similar compound, namely 4,6-bis­(4-fluoro­phen­yl)-2-phenyl-1*H*-indazol-3(2*H*)-one, see: Butcher *et al.* (2011 ▶). For information on graph-set motifs, see: Bernstein *et al.* (1995 ▶). For information on ring-puckering parameters, see: Cremer & Pople (1975 ▶).
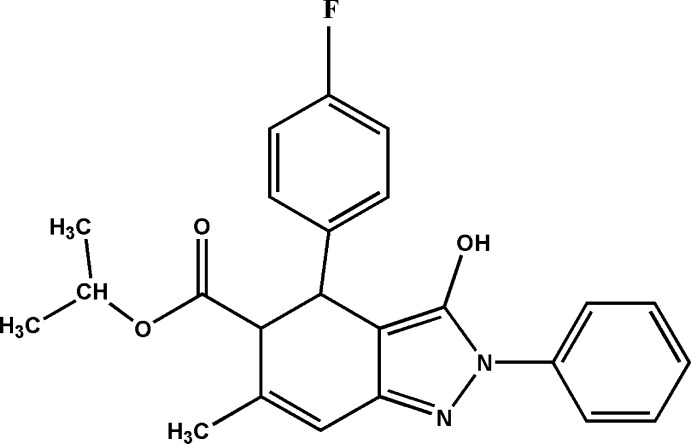


## Experimental

### 

#### Crystal data

C_24_H_23_FN_2_O_3_
*M* *_r_* = 406.44
Monoclinic, 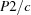

*a* = 17.640 (1) Å
*b* = 11.0295 (6) Å
*c* = 11.3791 (6) Å
β = 99.133 (1)°
*V* = 2185.9 (2) Å^3^
*Z* = 4
Mo *K*α radiation
μ = 0.09 mm^−1^
*T* = 293 K
0.35 × 0.25 × 0.25 mm

#### Data collection

Bruker SMART APEX CCD area-detector diffractometer
20548 measured reflections
3843 independent reflections
2970 reflections with *I* > 2σ(*I*)
*R*_int_ = 0.034

#### Refinement

*R*[*F* ^2^ > 2σ(*F* ^2^)] = 0.051
*wR*(*F* ^2^) = 0.135
*S* = 1.03
3843 reflections
279 parameters
H-atom parameters constrained
Δρ_max_ = 0.34 e Å^−3^
Δρ_min_ = −0.22 e Å^−3^

### 

Data collection: *SMART* (Bruker, 2001 ▶); cell refinement: *SAINT* (Bruker, 2001 ▶); data reduction: *SAINT*; program(s) used to solve structure: *SIR92* (Altomare *et al.*, 1994 ▶); program(s) used to refine structure: *SHELXL97* (Sheldrick, 2008 ▶); molecular graphics: *ORTEP-3* (Farrugia, 1999 ▶) and *PLATON* (Spek, 2009 ▶); software used to prepare material for publication: *SHELXL97*, *PLATON* and *publCIF* (Westrip, 2010 ▶).

## Supplementary Material

Crystal structure: contains datablock(s) I, global. DOI: 10.1107/S1600536812039955/su2500sup1.cif

Structure factors: contains datablock(s) I. DOI: 10.1107/S1600536812039955/su2500Isup2.hkl

Supplementary material file. DOI: 10.1107/S1600536812039955/su2500Isup3.cml

Additional supplementary materials:  crystallographic information; 3D view; checkCIF report

## Figures and Tables

**Table 1:** Hydrogen-bond geometry (Å, °) *Cg*1 is the centroid of the N1/N2/C3/C8/C9 ring.

*D*—H⋯*A*	*D*—H	H⋯*A*	*D*⋯*A*	*D*—H⋯*A*
O1—H1⋯N1^i^	0.82	1.82	2.6143 (18)	162
C22—H22⋯O52^ii^	0.93	2.56	3.229 (6)	129
C24—H24⋯*Cg*1^iii^	0.93	2.77	3.694 (3)	174

Symmetry codes: (i) 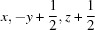; (ii) 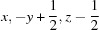; (iii) 
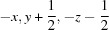
.

**Fig. 1.**
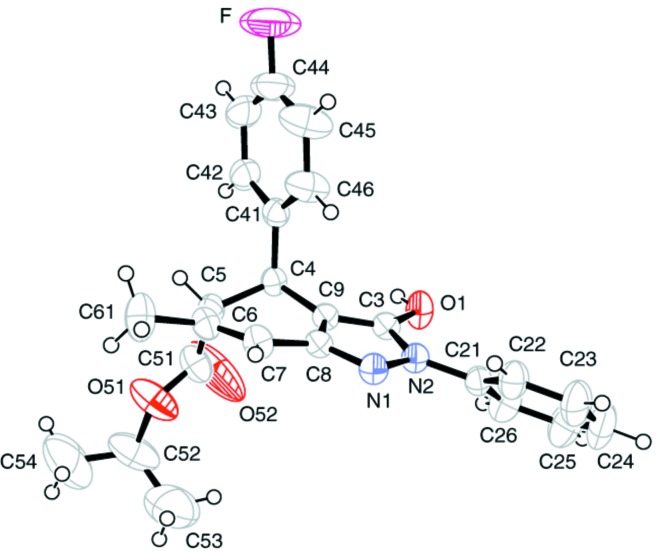
View of the molecule structure of the title molecule, showing the atom-labelling. Displacement ellipsoids are drawn at the 50% probability level. Only the major component of the disordered atom O52 is shown.

**Fig. 2.**
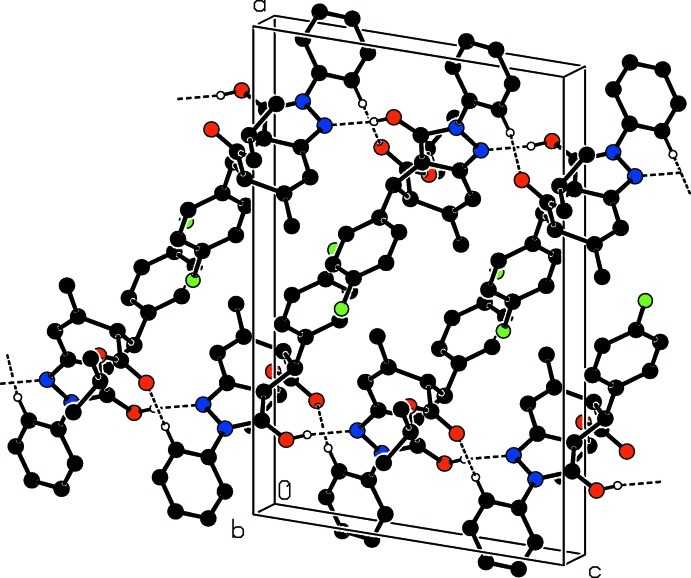
A view along the b axis of the crystal packing diagram of the title compound. Dashed lines indicate hydrogen bonds (see Table 1 for details).

## supplementary crystallographic information

### Comment

Indazole derivatives possess a wide spectrum of pharmacological activities, such as analgestic, anti-inflammatory, anti-depressant, anti-hypertensive, anti-viral and anti-cancer (Jain *et al.*, 1987; Palazzo *et al.*, 1966; Popat *et al.*, 2003; Beylin *et al.*, 1991; George *et al.*, 1998; Roman, 1990). In the view of these important attributes, the title compound was synthesized and its crystal structure is described herein.

In the title molecule, Fig. 1, the cyclohexene ring adopts a scew-boat conformation with puckering parameters: Q = 0.391 (2) Å,θ = 61.7 (3)° and φ = 28.7 (3)° (Cremer & Pople, 1975). The methyl group attached at C6 is substituted in the β equitorial position [C8—C7—C6—C61 = 176.7 (2)°]. The pyrazole ring (N1/N2/C3/C8/C9) is almost planar with a maximum deviation of 0.010 (2) Å for atom N2. This five membered ring and the attached phenyl (C21-C26) ring make a dihedral angle of 37.51 (12)°. The same phenyl ring makes a dihedral angle of 57.77 (13)° with the fluorophenyl ring (C41-C46). This is similar to the situation in a related structure, 4,6-Bis(4-fluorophenyl)-2-phenyl-1H-indazol-3(2H)-one (Butcher *et al.*, 2011), where the same angle is 57.69 (10)°.

In the crystal, molecules are linked by O—H···N and weak C— H···O hydrogen bonds, forming chains with C(5) and C(10) graph-set motifs (Bernstein *et al.*, 1995) [Table 1 and Fig. 2]. There are also C-H···π interactions present (Table 1).

### Experimental

A mixture of isopropyl acetoacetate(14.4 g, 100 mmol), 4-fluorobenzaldehyde(6.2 g, 50 mmol) and methylamine(1.55 g, 50 mmol) in ethanol(50 ml) was heated to boiling. The reaction mixture was kept overnight and the solid that seperated out was filtered off and purified by recrystallization from ethanol. 9.5 g (25 mmmol) of this product, r(2),c(4)-bis(isopropoxycarbonyl)-c(5)-hydroxy-t(5)- methyl-t(3)-(4'-fluorophenyl)cyclohexanone (yeild 14.8 g, 78%, M.p. 452 K), was dissolved in acetic acid and after the addition of phenylhydrazine (4.3 g, 40 mmol) in the presence of sodium acetate (2.9 g, 50 mmol), the reaction mixture was refluxed for 4 h. The solution was cooled and then poured into crushed ice. The precipitated solid was filtered off by suction (yield 70%, M.p. 493 K) and recrystallized from ethanol giving block-like colourless crystals.

### Refinement

The isopropycarboxylate group undergoes considerable thermal motion. Atom O52 was refined as disordered over two positions (O52/O52') and had a final refined occupancy ratio of 0.724 (15):0.276 (15). The C-bound H-atoms were included in calculated positions and treated as riding atoms: O-H = 0.82 Å, C-H = 0.93, 0.98 and 0.96 Å for CH(aromatic), CH and CH_3_ H-atoms, respectively, with U_iso_(H) = k × U_eq_(parent C-atom), where k = 1.5 for OH and CH_3_ H-atoms and = 1.2 for other H-atoms. Reflection 1 0 0 was partially obscured by the beam stop and was omitted.

### Crystal data

**Table:**

C_24_H_23_FN_2_O_3_	*F*(000) = 856
*M**_r_* = 406.44	*D*_x_ = 1.235 Mg m^−^^3^
Monoclinic, *P*2/*c*	Mo *K*α radiation, λ = 0.71073 Å
Hall symbol: -P 2ybc	Cell parameters from 3844 reflections
*a* = 17.640 (1) Å	θ = 2–25°
*b* = 11.0295 (6) Å	µ = 0.09 mm^−^^1^
*c* = 11.3791 (6) Å	*T* = 293 K
β = 99.133 (1)°	Block, colourless
*V* = 2185.9 (2) Å^3^	0.35 × 0.25 × 0.25 mm
*Z* = 4	

### Data collection

**Table:**

Bruker SMART APEX CCD area-detector diffractometer	2970 reflections with *I* > 2σ(*I*)
Radiation source: fine-focus sealed tube	*R*_int_ = 0.034
Graphite monochromator	θ_max_ = 25.0°, θ_min_ = 2.2°
ω scan	*h* = −20→20
20548 measured reflections	*k* = −13→13
3843 independent reflections	*l* = −13→13

### Refinement

**Table:**

Refinement on *F*^2^	Primary atom site location: structure-invariant direct methods
Least-squares matrix: full	Secondary atom site location: difference Fourier map
*R*[*F*^2^ > 2σ(*F*^2^)] = 0.051	Hydrogen site location: inferred from neighbouring sites
*wR*(*F*^2^) = 0.135	H-atom parameters constrained
*S* = 1.03	*w* = 1/[σ^2^(*F*_o_^2^) + (0.0586*P*)^2^ + 0.9373*P*] where *P* = (*F*_o_^2^ + 2*F*_c_^2^)/3
3843 reflections	(Δ/σ)_max_ < 0.001
279 parameters	Δρ_max_ = 0.34 e Å^−^^3^
0 restraints	Δρ_min_ = −0.22 e Å^−^^3^

### Special details

**Table:**

Geometry. All e.s.d.'s (except the e.s.d. in the dihedral angle between two l.s. planes) are estimated using the full covariance matrix. The cell e.s.d.'s are taken into account individually in the estimation of e.s.d.'s in distances, angles and torsion angles; correlations between e.s.d.'s in cell parameters are only used when they are defined by crystal symmetry. An approximate (isotropic) treatment of cell e.s.d.'s is used for estimating e.s.d.'s involving l.s. planes.
Refinement. Refinement of *F*^2^ against ALL reflections. The weighted *R*-factor *wR* and goodness of fit *S* are based on *F*^2^, conventional *R*-factors *R* are based on *F*, with *F* set to zero for negative *F*^2^. The threshold expression of *F*^2^ > σ(*F*^2^) is used only for calculating *R*-factors(gt) *etc*. and is not relevant to the choice of reflections for refinement. *R*-factors based on *F*^2^ are statistically about twice as large as those based on *F*, and *R*- factors based on ALL data will be even larger.

### Fractional atomic coordinates and isotropic or equivalent isotropic displacement parameters (Å^2^)

**Table:**

	*x*	*y*	*z*	*U*_iso_*/*U*_eq_	Occ. (<1)
F	0.55203 (11)	0.4061 (2)	0.2222 (2)	0.1378 (9)	
O1	0.14456 (9)	0.32293 (13)	0.05959 (11)	0.0505 (4)	
H1	0.1609	0.2941	0.1252	0.076*	
O51	0.26507 (13)	−0.19468 (16)	−0.01810 (17)	0.0885 (6)	
O52	0.2223 (4)	−0.1001 (5)	0.1280 (9)	0.116 (2)	0.724 (15)
O52'	0.1999 (12)	−0.0778 (13)	0.0673 (18)	0.116 (2)	0.276 (15)
N2	0.14598 (9)	0.28868 (14)	−0.13981 (12)	0.0369 (4)	
N1	0.18599 (9)	0.22532 (14)	−0.21440 (12)	0.0386 (4)	
C3	0.17400 (10)	0.26286 (16)	−0.02333 (14)	0.0356 (4)	
C4	0.28913 (11)	0.12354 (17)	0.07402 (16)	0.0376 (4)	
H4	0.2638	0.1076	0.1431	0.045*	
C5	0.31565 (12)	0.00027 (17)	0.02803 (17)	0.0430 (5)	
H5	0.3642	−0.0202	0.0788	0.052*	
C6	0.33235 (12)	0.00401 (18)	−0.09910 (18)	0.0453 (5)	
C7	0.29229 (12)	0.07767 (18)	−0.17831 (17)	0.0443 (5)	
H7	0.3004	0.0767	−0.2571	0.053*	
C8	0.23623 (10)	0.15914 (16)	−0.14217 (15)	0.0362 (4)	
C9	0.23134 (10)	0.17912 (16)	−0.02211 (15)	0.0351 (4)	
C21	0.08425 (11)	0.36638 (17)	−0.18736 (16)	0.0396 (4)	
C22	0.09024 (13)	0.4340 (2)	−0.28730 (18)	0.0538 (6)	
H22	0.1342	0.4292	−0.3226	0.065*	
C23	0.03079 (16)	0.5085 (2)	−0.3344 (2)	0.0722 (7)	
H23	0.0347	0.5539	−0.4021	0.087*	
C24	−0.03366 (17)	0.5169 (3)	−0.2835 (3)	0.0826 (9)	
H24	−0.0738	0.5671	−0.3165	0.099*	
C25	−0.03905 (15)	0.4505 (3)	−0.1829 (3)	0.0794 (8)	
H25	−0.0828	0.4571	−0.1472	0.095*	
C26	0.01950 (13)	0.3744 (2)	−0.1345 (2)	0.0589 (6)	
H26	0.0154	0.3289	−0.0669	0.071*	
C41	0.35810 (11)	0.20446 (17)	0.11368 (17)	0.0424 (5)	
C42	0.40331 (13)	0.1842 (2)	0.2224 (2)	0.0575 (6)	
H42	0.3898	0.1232	0.2715	0.069*	
C43	0.46809 (15)	0.2525 (3)	0.2599 (3)	0.0768 (8)	
H43	0.4980	0.2382	0.3335	0.092*	
C44	0.48710 (16)	0.3400 (3)	0.1879 (3)	0.0832 (9)	
C45	0.44411 (19)	0.3651 (3)	0.0811 (3)	0.0933 (10)	
H45	0.4582	0.4270	0.0335	0.112*	
C46	0.37878 (15)	0.2966 (2)	0.0444 (2)	0.0690 (7)	
H46	0.3485	0.3134	−0.0284	0.083*	
C51	0.25938 (15)	−0.0996 (2)	0.0456 (2)	0.0570 (6)	
C52	0.2159 (2)	−0.3005 (3)	−0.0069 (3)	0.0973 (11)	
H52	0.2039	−0.3045	0.0742	0.117*	
C53	0.1431 (3)	−0.2891 (4)	−0.0942 (4)	0.1460 (17)	
H53A	0.1171	−0.2157	−0.0786	0.219*	
H53B	0.1105	−0.3574	−0.0861	0.219*	
H53C	0.1550	−0.2868	−0.1736	0.219*	
C54	0.2622 (3)	−0.4083 (3)	−0.0300 (5)	0.176 (2)	
H54A	0.2719	−0.4060	−0.1106	0.264*	
H54B	0.2344	−0.4809	−0.0177	0.264*	
H54C	0.3101	−0.4076	0.0235	0.264*	
C61	0.39422 (15)	−0.0780 (2)	−0.1293 (2)	0.0732 (8)	
H61A	0.3978	−0.0699	−0.2122	0.110*	
H61B	0.3821	−0.1605	−0.1127	0.110*	
H61C	0.4424	−0.0562	−0.0823	0.110*	

### Atomic displacement parameters (Å^2^)

**Table:**

	*U*^11^	*U*^22^	*U*^33^	*U*^12^	*U*^13^	*U*^23^
F	0.0942 (13)	0.1364 (18)	0.175 (2)	−0.0579 (13)	−0.0032 (13)	−0.0562 (16)
O1	0.0724 (10)	0.0546 (9)	0.0250 (7)	0.0222 (7)	0.0091 (6)	0.0010 (6)
O51	0.1363 (17)	0.0548 (11)	0.0841 (13)	−0.0338 (11)	0.0475 (12)	−0.0155 (9)
O52	0.190 (5)	0.060 (2)	0.130 (5)	−0.038 (2)	0.120 (5)	−0.015 (3)
O52'	0.190 (5)	0.060 (2)	0.130 (5)	−0.038 (2)	0.120 (5)	−0.015 (3)
N2	0.0446 (9)	0.0414 (9)	0.0246 (7)	0.0058 (7)	0.0059 (6)	0.0001 (6)
N1	0.0469 (9)	0.0430 (9)	0.0267 (8)	0.0047 (7)	0.0086 (7)	−0.0009 (7)
C3	0.0464 (11)	0.0372 (10)	0.0232 (9)	−0.0012 (8)	0.0061 (8)	−0.0001 (7)
C4	0.0446 (11)	0.0376 (10)	0.0310 (9)	0.0030 (8)	0.0069 (8)	0.0035 (8)
C5	0.0473 (11)	0.0386 (11)	0.0419 (11)	0.0041 (9)	0.0038 (9)	0.0050 (8)
C6	0.0508 (12)	0.0374 (11)	0.0502 (12)	0.0023 (9)	0.0156 (10)	−0.0006 (9)
C7	0.0554 (12)	0.0447 (11)	0.0358 (10)	0.0026 (10)	0.0167 (9)	−0.0001 (9)
C8	0.0424 (10)	0.0357 (10)	0.0308 (9)	−0.0002 (8)	0.0069 (8)	0.0005 (8)
C9	0.0421 (10)	0.0347 (10)	0.0278 (9)	0.0000 (8)	0.0039 (8)	0.0007 (7)
C21	0.0466 (11)	0.0391 (10)	0.0318 (9)	0.0043 (9)	0.0020 (8)	−0.0020 (8)
C22	0.0616 (14)	0.0564 (13)	0.0436 (12)	0.0109 (11)	0.0086 (10)	0.0102 (10)
C23	0.0874 (19)	0.0681 (17)	0.0581 (15)	0.0247 (15)	0.0020 (14)	0.0201 (12)
C24	0.0769 (19)	0.0825 (19)	0.084 (2)	0.0399 (16)	−0.0019 (15)	0.0140 (16)
C25	0.0602 (16)	0.097 (2)	0.0829 (19)	0.0294 (15)	0.0176 (14)	0.0055 (17)
C26	0.0548 (13)	0.0712 (16)	0.0519 (13)	0.0120 (12)	0.0126 (11)	0.0084 (11)
C41	0.0474 (11)	0.0405 (11)	0.0393 (10)	0.0046 (9)	0.0064 (9)	−0.0047 (9)
C42	0.0580 (14)	0.0562 (14)	0.0531 (13)	0.0080 (11)	−0.0070 (11)	−0.0061 (11)
C43	0.0652 (17)	0.0789 (19)	0.0766 (18)	0.0082 (15)	−0.0182 (14)	−0.0249 (16)
C44	0.0624 (17)	0.084 (2)	0.100 (2)	−0.0204 (15)	0.0017 (16)	−0.0396 (19)
C45	0.106 (2)	0.086 (2)	0.089 (2)	−0.0484 (19)	0.0201 (19)	−0.0070 (17)
C46	0.0818 (17)	0.0691 (16)	0.0535 (14)	−0.0267 (14)	0.0024 (12)	0.0025 (12)
C51	0.0759 (16)	0.0414 (12)	0.0585 (14)	0.0012 (11)	0.0251 (12)	0.0012 (10)
C52	0.142 (3)	0.0652 (19)	0.088 (2)	−0.047 (2)	0.029 (2)	−0.0090 (16)
C53	0.155 (4)	0.104 (3)	0.172 (4)	−0.040 (3)	0.003 (3)	−0.014 (3)
C54	0.198 (5)	0.054 (2)	0.274 (7)	−0.019 (3)	0.033 (5)	−0.004 (3)
C61	0.0776 (17)	0.0674 (16)	0.0804 (18)	0.0276 (14)	0.0305 (14)	0.0079 (14)

### Geometric parameters (Å, º)

**Table:**

F—C44	1.361 (3)	C23—H23	0.9300
O1—C3	1.325 (2)	C24—C25	1.375 (4)
O1—H1	0.8200	C24—H24	0.9300
O51—C51	1.288 (3)	C25—C26	1.376 (3)
O51—C52	1.472 (3)	C25—H25	0.9300
O52—C51	1.225 (7)	C26—H26	0.9300
O52'—C51	1.142 (18)	C41—C46	1.371 (3)
N2—C3	1.369 (2)	C41—C42	1.380 (3)
N2—N1	1.377 (2)	C42—C43	1.378 (4)
N2—C21	1.423 (2)	C42—H42	0.9300
N1—C8	1.328 (2)	C43—C44	1.343 (4)
C3—C9	1.368 (3)	C43—H43	0.9300
C4—C9	1.502 (2)	C44—C45	1.354 (4)
C4—C41	1.519 (3)	C45—C46	1.386 (4)
C4—C5	1.555 (3)	C45—H45	0.9300
C4—H4	0.9800	C46—H46	0.9300
C5—C51	1.517 (3)	C52—C54	1.489 (6)
C5—C6	1.522 (3)	C52—C53	1.499 (5)
C5—H5	0.9800	C52—H52	0.9800
C6—C7	1.330 (3)	C53—H53A	0.9600
C6—C61	1.499 (3)	C53—H53B	0.9600
C7—C8	1.444 (3)	C53—H53C	0.9600
C7—H7	0.9300	C54—H54A	0.9600
C8—C9	1.400 (2)	C54—H54B	0.9600
C21—C26	1.376 (3)	C54—H54C	0.9600
C21—C22	1.378 (3)	C61—H61A	0.9600
C22—C23	1.372 (3)	C61—H61B	0.9600
C22—H22	0.9300	C61—H61C	0.9600
C23—C24	1.358 (4)		
			
C3—O1—H1	109.5	C25—C26—H26	120.5
C51—O51—C52	119.8 (2)	C46—C41—C42	117.8 (2)
C3—N2—N1	110.45 (14)	C46—C41—C4	122.59 (18)
C3—N2—C21	129.06 (15)	C42—C41—C4	119.56 (19)
N1—N2—C21	120.48 (14)	C43—C42—C41	121.5 (2)
C8—N1—N2	104.75 (13)	C43—C42—H42	119.3
O1—C3—C9	134.69 (16)	C41—C42—H42	119.3
O1—C3—N2	117.61 (16)	C44—C43—C42	118.6 (3)
C9—C3—N2	107.64 (15)	C44—C43—H43	120.7
C9—C4—C41	113.30 (15)	C42—C43—H43	120.7
C9—C4—C5	108.49 (15)	C43—C44—C45	122.4 (3)
C41—C4—C5	110.14 (15)	C43—C44—F	119.3 (3)
C9—C4—H4	108.3	C45—C44—F	118.3 (3)
C41—C4—H4	108.3	C44—C45—C46	118.7 (3)
C5—C4—H4	108.3	C44—C45—H45	120.7
C51—C5—C6	111.98 (17)	C46—C45—H45	120.7
C51—C5—C4	110.64 (16)	C41—C46—C45	121.0 (3)
C6—C5—C4	114.21 (15)	C41—C46—H46	119.5
C51—C5—H5	106.5	C45—C46—H46	119.5
C6—C5—H5	106.5	O52'—C51—O52	38.2 (9)
C4—C5—H5	106.5	O52'—C51—O51	116.8 (8)
C7—C6—C61	122.76 (19)	O52—C51—O51	121.7 (3)
C7—C6—C5	119.90 (17)	O52'—C51—C5	121.2 (7)
C61—C6—C5	117.34 (18)	O52—C51—C5	123.0 (3)
C6—C7—C8	120.09 (17)	O51—C51—C5	114.0 (2)
C6—C7—H7	120.0	O51—C52—C54	105.7 (3)
C8—C7—H7	120.0	O51—C52—C53	109.3 (3)
N1—C8—C9	112.28 (16)	C54—C52—C53	112.7 (3)
N1—C8—C7	125.88 (16)	O51—C52—H52	109.7
C9—C8—C7	121.81 (17)	C54—C52—H52	109.7
C3—C9—C8	104.85 (15)	C53—C52—H52	109.7
C3—C9—C4	134.26 (16)	C52—C53—H53A	109.5
C8—C9—C4	120.43 (16)	C52—C53—H53B	109.5
C26—C21—C22	120.27 (19)	H53A—C53—H53B	109.5
C26—C21—N2	120.68 (18)	C52—C53—H53C	109.5
C22—C21—N2	119.04 (18)	H53A—C53—H53C	109.5
C23—C22—C21	119.5 (2)	H53B—C53—H53C	109.5
C23—C22—H22	120.2	C52—C54—H54A	109.5
C21—C22—H22	120.2	C52—C54—H54B	109.5
C24—C23—C22	120.9 (2)	H54A—C54—H54B	109.5
C24—C23—H23	119.5	C52—C54—H54C	109.5
C22—C23—H23	119.5	H54A—C54—H54C	109.5
C23—C24—C25	119.5 (2)	H54B—C54—H54C	109.5
C23—C24—H24	120.3	C6—C61—H61A	109.5
C25—C24—H24	120.3	C6—C61—H61B	109.5
C24—C25—C26	120.8 (2)	H61A—C61—H61B	109.5
C24—C25—H25	119.6	C6—C61—H61C	109.5
C26—C25—H25	119.6	H61A—C61—H61C	109.5
C21—C26—C25	119.1 (2)	H61B—C61—H61C	109.5
C21—C26—H26	120.5		
			
C3—N2—N1—C8	1.81 (19)	N1—N2—C21—C22	−38.0 (3)
C21—N2—N1—C8	−177.34 (16)	C26—C21—C22—C23	−0.7 (3)
N1—N2—C3—O1	175.77 (16)	N2—C21—C22—C23	179.5 (2)
C21—N2—C3—O1	−5.2 (3)	C21—C22—C23—C24	0.3 (4)
N1—N2—C3—C9	−1.8 (2)	C22—C23—C24—C25	0.5 (5)
C21—N2—C3—C9	177.29 (17)	C23—C24—C25—C26	−1.1 (5)
C9—C4—C5—C51	−83.40 (19)	C22—C21—C26—C25	0.1 (4)
C41—C4—C5—C51	152.07 (17)	N2—C21—C26—C25	180.0 (2)
C9—C4—C5—C6	44.0 (2)	C24—C25—C26—C21	0.7 (4)
C41—C4—C5—C6	−80.5 (2)	C9—C4—C41—C46	−21.0 (3)
C51—C5—C6—C7	94.1 (2)	C5—C4—C41—C46	100.8 (2)
C4—C5—C6—C7	−32.6 (3)	C9—C4—C41—C42	160.39 (18)
C51—C5—C6—C61	−85.5 (2)	C5—C4—C41—C42	−77.9 (2)
C4—C5—C6—C61	147.7 (2)	C46—C41—C42—C43	−1.2 (3)
C61—C6—C7—C8	−176.7 (2)	C4—C41—C42—C43	177.5 (2)
C5—C6—C7—C8	3.7 (3)	C41—C42—C43—C44	−0.2 (4)
N2—N1—C8—C9	−1.2 (2)	C42—C43—C44—C45	1.4 (5)
N2—N1—C8—C7	−179.28 (17)	C42—C43—C44—F	−178.3 (2)
C6—C7—C8—N1	−170.79 (19)	C43—C44—C45—C46	−0.9 (5)
C6—C7—C8—C9	11.3 (3)	F—C44—C45—C46	178.7 (3)
O1—C3—C9—C8	−176.0 (2)	C42—C41—C46—C45	1.6 (4)
N2—C3—C9—C8	1.0 (2)	C4—C41—C46—C45	−177.0 (2)
O1—C3—C9—C4	−4.1 (4)	C44—C45—C46—C41	−0.6 (5)
N2—C3—C9—C4	172.85 (19)	C52—O51—C51—O52'	−33.0 (12)
N1—C8—C9—C3	0.2 (2)	C52—O51—C51—O52	10.8 (7)
C7—C8—C9—C3	178.33 (17)	C52—O51—C51—C5	177.8 (2)
N1—C8—C9—C4	−173.10 (16)	C6—C5—C51—O52'	−114.5 (13)
C7—C8—C9—C4	5.1 (3)	C4—C5—C51—O52'	14.1 (13)
C41—C4—C9—C3	−80.1 (3)	C6—C5—C51—O52	−160.0 (6)
C5—C4—C9—C3	157.3 (2)	C4—C5—C51—O52	−31.3 (7)
C41—C4—C9—C8	90.8 (2)	C6—C5—C51—O51	33.2 (3)
C5—C4—C9—C8	−31.8 (2)	C4—C5—C51—O51	161.8 (2)
C3—N2—C21—C26	−36.8 (3)	C51—O51—C52—C54	−148.1 (3)
N1—N2—C21—C26	142.2 (2)	C51—O51—C52—C53	90.4 (4)
C3—N2—C21—C22	143.1 (2)		

### Hydrogen-bond geometry (Å, º)

Cg1 is the centroid of the N1/N2/C3/C8/C9 ring.

**Table:**

*D*—H···*A*	*D*—H	H···*A*	*D*···*A*	*D*—H···*A*
O1—H1···N1^i^	0.82	1.82	2.6143 (18)	162
C22—H22···O52^ii^	0.93	2.56	3.229 (6)	129
C24—H24···*Cg*1^iii^	0.93	2.77	3.694 (3)	174

Symmetry codes: (i) *x*, −*y*+1/2, *z*+1/2; (ii) *x*, −*y*+1/2, *z*−1/2; (iii) −*x*, *y*+1/2, −*z*−1/2.
